# *Anopheles sacharovi* in Italy: first record of the historical malaria vector after over 50 years

**DOI:** 10.1186/s13071-024-06252-2

**Published:** 2024-04-10

**Authors:** Donato Antonio Raele, Francesco Severini, Luciano Toma, Michela Menegon, Daniela Boccolini, Giovanni Tortorella, Marco Di Luca, Maria Assunta Cafiero

**Affiliations:** 1 Istituto Zooprofilattico Sperimentale della Puglia e Della Basilicata, Via Manfredonia, 20, 71121 Foggia, Italia; 2 Dipartimento di Malattie Infettive, Reparto di Malattie Trasmesse da Vettori, Istituto Superiore di Sanità, Viale Regina Elena, 299, 00161 Rome, Italia; 3 Azienda Sanitaria Nazionale (ASL), Servizio Veterinario Sanità Animale, Viale Don Minzoni N. 8, 73100 Lecce, Italia

**Keywords:** *Anopheles sacharovi*, Malaria, Italy, qPCR, *Anopheles maculipennis* complex, Residual anophelism

## Abstract

**Background:**

*Anopheles sacharovi*, a member of the *Anopheles maculipennis* complex, was a historical malaria vector in Italy, no longer found since the last report at the end of 1960s. In September 2022, within the Surveillance Project for the residual anophelism, a single specimen of *An. maculipennis* sensu lato collected in Lecce municipality (Apulia region) was molecularly identified as *An. sacharovi*. This record led to implement a targeted entomological survey in September 2023.

**Methods:**

Investigation was conducted in the areas around the first discovery, focusing on animal farms, riding stables and potential breeding sites. Adult and immature mosquitoes were collected, using active search or traps, in several natural and rural sites. Mosquitoes belonging to *An. maculipennis* complex were identified morphologically and molecularly by a home-made routine quantitative polymerase chain reaction (qPCR) assay, developed specifically for the rapid identification of *An. labranchiae*, and, when necessary, by amplification and sequencing of the ITS-2 molecular marker.

**Results:**

Out of the 11 sites investigated, 6 were positive for *Anopheles* presence. All 20 *An. maculipennis* s.l. (7 adults, 10 larvae and 3 pupae) collected in the areas were identified as *An. sacharovi* by ITS-2 sequencing.

**Conclusions:**

The discovery of *An. sacharovi*, considered to have disappeared from Italy for over 50 years, has a strong health relevance and impact, highlighting an increase in the receptivity of the southern areas. As imported malaria cases in European countries are reported every year, the risk of *Plasmodium* introduction by gametocyte carriers among travellers from endemic countries should be taken into greater consideration. Our findings allow rethinking and building new models for the prediction and expansion of introduced malaria. Furthermore, to prevent the risk of reintroduction of the disease, the need to strengthen the surveillance of residual anophelism throughout the South should be considered.

**Graphical Abstract:**

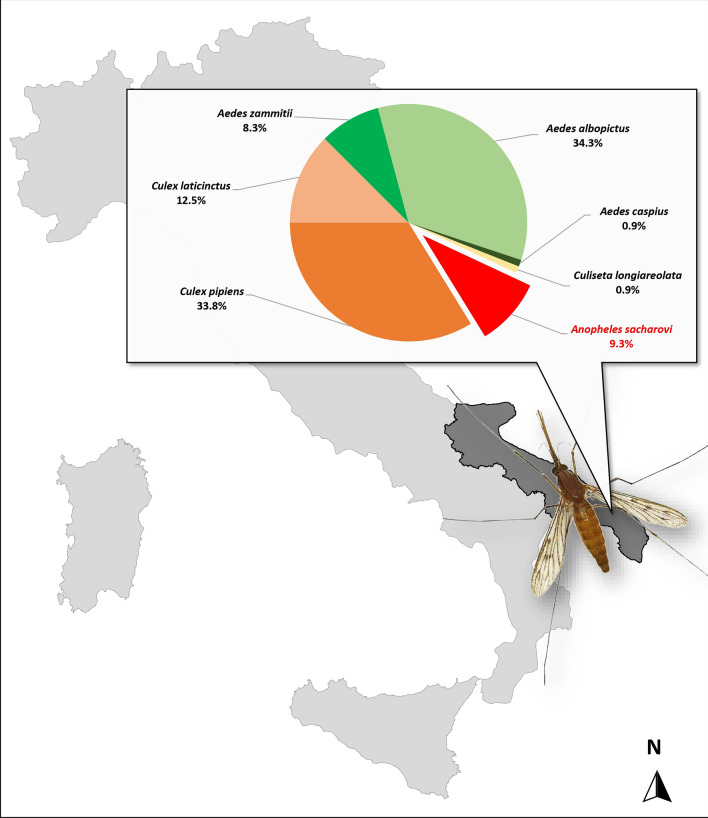

## Background

Malaria was endemic in Italy until the middle of the last century. Human cases, mainly due to *Plasmodium falciparum* and *P. vivax*, were widespread especially along the coastal plains, and before the decisive interventions of reclamation and the launch of the National Anti-malarial Campaign, the disease affected hundreds of people every year [1]. Although Italy was officially declared malaria free by the World Health Organization (WHO) in 1970, since then a few sporadic cases of non-imported malaria have been recorded. Among the most recent cases, four non-travel related malaria cases occurred in Ginosa (Taranto, Apulia region) in October 2017. Integrated entomological surveys were performed, and the presence of the potential malaria vector, *Anopheles labranchiae*, was recorded in the involved areas [2]. In Italy, there are currently six anopheline sibling species belonging to the *Anopheles maculipennis* Meigen complex that cannot be distinguished morphologically, including *An. atroparvus* Van Thiel, 1927, *An. labranchiae* Falleroni, 1926, *An. maculipennis* sensu stricto Meigen, 1818, *An. melanoon* Hackett, 1934, *An. messeae* Falleroni, 1926, and *An. daciae* species inquirenda Linton, Nicolescu & Harbach, 2004 [3, 4]. A seventh sibling taxon, *Anopheles sacharovi* Favre, 1903, once widely distributed throughout the country, progressively disappeared probably because of the progressive modification of its larval habitats [5–10]. Among the *An. maculipennis* complex, *An. labranchiae* and *An. sacharovi* were historically considered the two most competent malaria vectors in Italy, always associates with endemic malaria and involved in *P. falciparum* and *P. vivax* transmission [11–13]. These two species were common and sometimes found in sympatry in Central and South Italy, including the Apulia region [10–14]. *Anopheles labranchiae* is still abundant and widespread in the region, especially in the Gargano, as recently documented [12, 15]. Contrarily, despite two doubtful findings reported in Northern and Central Italy [16, 17], *An. sacharovi* has not been found since the last report in the late 1960s. This mosquito thrived in several rural areas, mostly along the north and southwestern coastal plains and in northern Sardinia [5]. Although exophagic activity has been described for this species, it commonly exhibited endophagic and endophilic behaviour, resting in human dwellings, flooded basements and animal shelters where it could bite both day and night [18, 19]. This paper describes the investigation that led to the rediscovery of *An. sacharovi* in Italy, specifically in the Apulia region.

## Methods

**Fig. 1:**
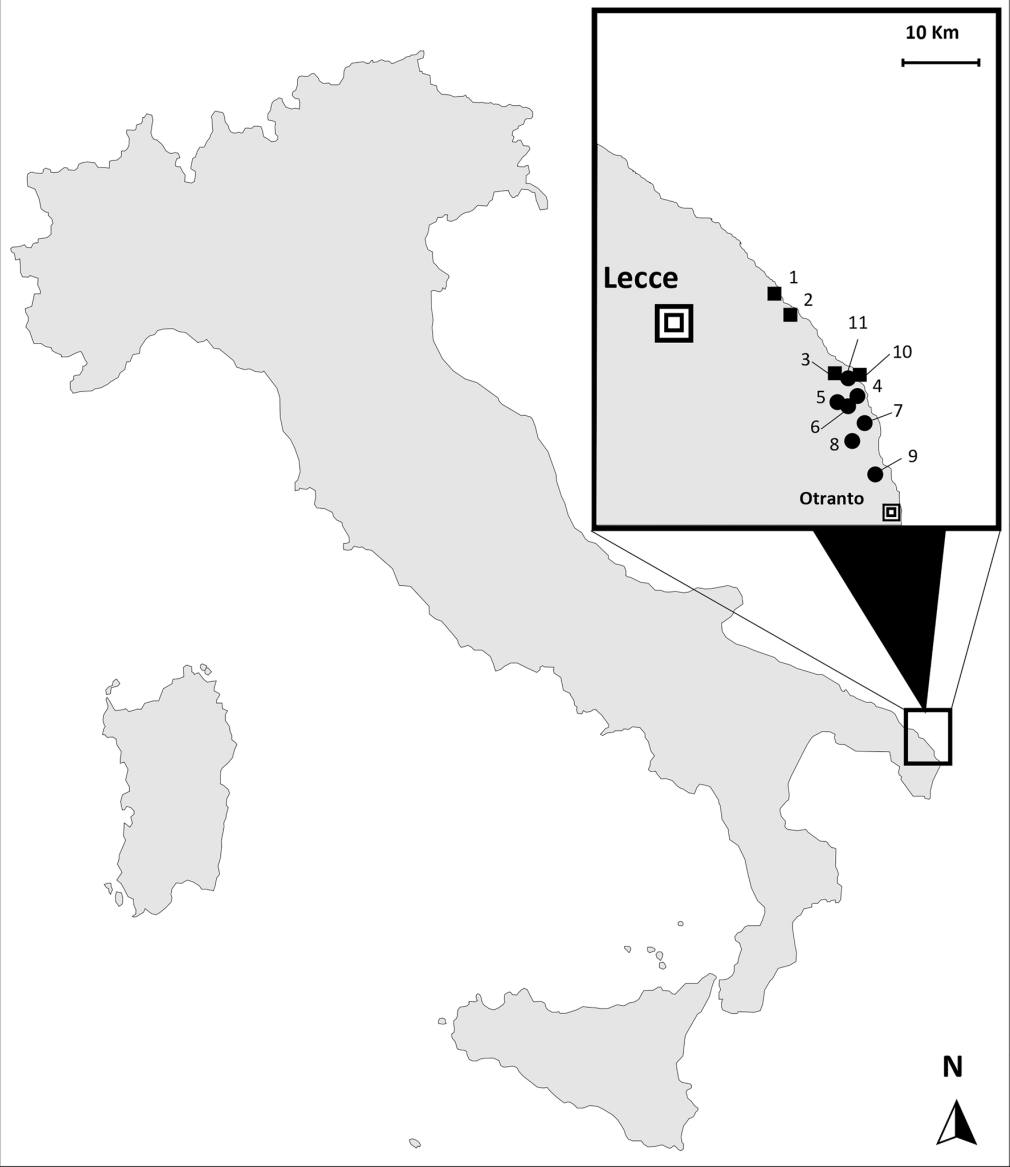
Mosquito collection sites in the 2-year period, 2022–2023. Black dot: adult collection sites; black square: larval breeding sites

In 2022, the Italian Ministry of Health funded a research project (RC IZSPB 06/2022) to monitor the presence and the extent of *An. maculipennis* complex mosquitoes in Apulia and Basilicata territories. The Centers for Disease Control light trap (CDC-LT) network was extended to the area of Lecce Province in southeastern Italy. During the year, only one *Anopheles* specimen was collected in early September, in a horse-riding stable, and it was morphologically identified as *An. maculipennis* s.l. according to Severini et al. [20]. The mosquito was subsequently analysed by a home-made qPCR, an assay developed for routine rapid screening of *An. labranchiae* identification, the most widespread species of the complex in the region. A primer set was designed to amplify a 95-bp fragment of nuclear ribosomal internal transcribed spacer 2 (ITS-2) of *An. labranchiae*. The reaction was carried out in a 20-µl reaction using the SsoAdvanced Universal ProbeMix (BIORAD) with primers L1: 5′-GGTCATCGTGAGGCGTTATC-3′ and R1: 5′-GCTAGGAGCCGGTCTTGTAT-3′ at concentrations of 0.5 µM each and the probe P1: FAM-5′-AAGCACTCGCTGCTGCGCGT-BHQ1′ at a concentration of 0.2 µM under the following conditions: 95 ℃ for 3 min, 30 cycles at 95 ℃ for 10 s and 60 ℃ for 20 s. The negative qPCR sample was tested by conventional PCR. ITS-2 amplicon was sequenced at Eurofins Genomics (Ebersberg, Germany) and analysed by National Center for Biotechnology Information’s (NCBI) Basic Local Alignment Search (BLAST) for the identification of mosquito species [21]. The resulting sequence was deposited in GenBank (accession no. OQ748043). In September 2023, the entomological investigation was extended from the positive horse-riding stable (site 6) to neighbouring coastal areas of Lecce Province, including ten other different sites. In Fig. 1, the study area with the geographical distribution of the visited sites, for both adult and larval collections, is shown. The collection sites were selected in areas characterized by the presence of several marshes, brackish water basins and natural lakes and farms with animals (cattle, horses, sheep and poultry). The adult collections were carried out in animal shelters and human dwellings, using manual or battery-powered aspirators (modified Katcha® Bug Buster Spider Vacuum Blue) or CDC light traps. In potential breeding sites (i.e. irrigation and drainage canals, streams, marshes, large ponds and other permanent water collections), larvae and pupae were collected using a 500-ml standard dipper. All mosquito specimens were morphologically identified [20] and, when necessary, screened by molecular tools for species detection. Rapid DNA extraction for a single specimen was performed using a microwave method [22]. *Culex* and *Aedes* species and all specimens belonging to the *An. maculipennis* complex caught in 2023 were directly tested by conventional PCR, according to Marinucci et al. [21]. *ITS-2* amplicons were sequenced at Eurofins Genomics (Ebersberg, Germany) and analysed using NCBI’s Basic Local Alignment Search (BLAST) for the identification of mosquito species.

## Results

The single *An. maculipennis* s.l. collected in September 2022, at site 6 (Lecce Province), was molecularly analysed and identified as *An. sacharovi* (GenBank accession no. OQ748043). During the subsequent 2023 entomological survey, a total of 216 mosquitoes (125 immatures and 91 adults) were captured and identified morphologically, when possible. For *Culex pipiens, Cx. laticinctus*, *Aedes mariae* complex and *An. maculipennis* complex, targeted molecular analyses allowed the exact identification of the different taxa. Seven *An. maculipennis* s.l. females were collected on four farms (sites 4, 5, 8 and 9) and in a human dwelling (site 11); only one larval breeding site was positive for *Anopheles* presence, with 10 larvae and 3 pupae detected (site 2). All *An. maculipennis* s.l. were molecularly analysed and identified as *An. sacharovi*. ITS2 sequences obtained shared 100% nucleotide sequence identity with the sequence from a single specimen collected in 2022 (GenBank accession no. OQ748043). Table 1 summarizes the result obtained in this study, showing all sites visited in 2023, the number of specimens collected and the mosquito species detected. Overall, seven species were identified: *An. sacharovi* (9.3%), *Cx. pipiens* (33.8%), *Cx. laticinctus* (12.5%), *Aedes zammitii* (8.3%), *Ae. albopictus* (34.3%), *Ae. caspius* (0.9%), *Culiseta longiareolata* (0.9%).

**Table 1:** Number of mosquito specimens collected, species and sites visited during the entomological survey carried out in September 2023

Species	Larvae/pupae	Adults	ID collection site
*Anopheles sacharovi*	13	7	2, 4, 5, 8, 9, 11
*Culex pipiens*	13	60	1, 4, 5, 6, 7
*Culex laticinctus*	6	21	4, 7, 8
*Aedes zammitii*	18	–	10
*Aedes albopictus*	74	–	4, 7, 8
*Aedes caspius*	–	2	3, 4
*Culiseta longiareolata*	1	1	4, 7

## Discussion

Even after the anti-malarial campaign following the Second World War, *An. labranchiae* and *An. sacharovi* were recorded in Apulia region, often found in sympatry [23]. However due to the diking and draining of marshes and retrodunal ponds, the use of insecticides, urbanization and pollution, many larval habitats, typical of *An. sacharovi*, have been progressively reduced, and the species was reported in the region until the late 1960s [14]. This heavy anthropic impact has contributed to the rarefaction of *An. sacharovi* in its distribution, perhaps confining small and limited mosquito populations in restricted natural sites. More recently, a reduction in anthropic pressure, the expansion of natural areas and the creation of new ones, together with other favourable climatic and environmental factors, could have contributed to the slow reconquest of territories and reappearance of the species in Apulia region. The record of *Anopheles algeriensis* Theobald, 1903, a potential secondary malaria vector, recently reported in Apulia, confirms how these changes can positively allow the *Anopheles* species to spread and/or recolonize some areas [14, 24]. The recolonization of areas with characteristics favourable to the larval development of *An. algeriensis* could be a positive signal of environmental requalification, which could also be beneficial for the spread of *An. sacharovi*. As supported by the literature, *An. sacharovi* can colonise different types of water collections, such as swamps, marshes, river margins, streams, pools and ditches, and can develop in both freshwater and brackish-salt water collections [18].

## Conclusions

Although research projects have often been short and discontinuous, in recent years the collaborative activities conducted by the Experimental Zooprophylactic Institute of Puglia and Basilicata and the Istituto Superiore di Sanità have been able to clearly document the presence of potential malaria vectors, such as *An. labranchiae*, *Anopheles superpictus* Grassi, 1899 and *An. algeriensis* in the Apulia and Basilicata regions [14, 24]. Therefore, after decades of entomological investigations, this notable rediscovery in the coastal areas of southeastern Italy allows us to reintegrate *An. sacharovi* in the Italian Culicidae fauna. Adults of *An. sacharovi* were found indoors, either resting in animal shelters or landing on a human during dusk. This highlights not only an endophagic and endophilic behaviour but also a certain degree of anthropophilia of the species. Furthermore, a typical natural breeding site was identified, completing the entomological investigation. Beyond the exceptionality of such findings, it is necessary to underline the importance of strengthening and maintaining constant surveillance of residual anophelism, especially in vulnerable areas where the occurrence of sporadic malaria transmission could be possible. Further investigations will need to assess the distribution and seasonal abundance of *An. sacharovi* along the southeastern coasts of Italy. However, our findings, confirmed by two point-in-time investigations in 2 consecutive years, represent a valid basis for rethinking and building new models for the prediction and expansion of introduced malaria and for reconsidering the receptivity of the studied areas to malaria to prevent the risk of reintroduction of the disease. Although vector densities do not currently appear to be epidemiologically relevant enough to pose a health threat, the conditions for a renewal of transmission in several Mediterranean countries still exist, as reported in Greece [25], and the occurrence of foci of introduced malaria (in particular by *P. vivax*) in some regions of our country should not to be underestimated [13].

## Data Availability

The datasets generated and analysed during the current study are available in the NCBI (GenBank) repository by accession no. OQ748043. https://www.ncbi.nlm.nih.gov/nuccore/OQ748043.1
